# Physiological and Expressional Regulation on Photosynthesis, Starch and Sucrose Metabolism Response to Waterlogging Stress in Peanut

**DOI:** 10.3389/fpls.2021.601771

**Published:** 2021-07-02

**Authors:** Ruier Zeng, Tingting Chen, Xinyue Wang, Jing Cao, Xi Li, Xueyu Xu, Lei Chen, Qing Xia, Yonglong Dong, Luping Huang, Leidi Wang, Jialei Zhang, Lei Zhang

**Affiliations:** ^1^College of Agriculture, South China Agricultural University, Guangzhou, China; ^2^Bio-Tech Research Center, Shandong Academy of Agricultural Science, Jinan, China

**Keywords:** *Arachis hypogaea* L., waterlogging stress, photosynthesis, starch and sucrose metabolism, yield

## Abstract

Waterlogging has negative effects on crop yield. Physiological and transcriptome data of two peanut cultivars [Zhongkaihua 1 (ZKH 1) and Huayu 39 (HY 39)] were studied under normal water supply and waterlogging stress for 5 or 10 days at the flowering stage. The results showed that the main stem height, the number of lateral branches, lateral branch length, and the stem diameter increased under waterlogging stress, followed by an increase in dry matter accumulation, which was correlated with the increase in the soil and plant analysis development (SPAD) and net photosynthetic rate (Pn) and the upregulation of genes related to porphyrin and chlorophyll metabolism and photosynthesis. However, the imbalance of the source–sink relationship under waterlogging was the main cause of yield loss, and waterlogging caused an increase in the sucrose and soluble sugar contents and a decrease in the starch content; it also decreased the activities of sucrose synthetase (SS) and sucrose phosphate synthetase (SPS), which may be due to the changes in the expression of genes related to starch and sucrose metabolism. However, the imbalance of the source–sink relationship led to the accumulation of photosynthate in the stems and leaves, which resulted in the decrease of the ratio of pod dry weight to total dry weight (PDW/TDW) and yield. Compared with ZKH 1, the PDW of HY 39 decreased more probably because more photosynthate accumulated in the stem and leaves of HY 39 and could not be effectively transported to the pod.

## Introduction

Peanut plants (*Arachis hypogaea* L.), which has been cultivated worldwide, is an important source of oil and protein for humans. This is a tetraploid cultivar, which derived from an ancient hybridization of two diploid peanut ancestors. Waterlogging is an abiotic stress factor that has a strong negative impact on the pod yield and kernel quality of peanut (Chen W. et al., 2016; Ye et al., 2018). Such is the case in southern China, a region characterized by a subtropical monsoon climate with high temperature and humidity and frequent rainfall in May and June every year (Chan and Zhou, 2005; Zhou et al., 2013), during which peanut plants are in the middle or late growth stages of the spring cropping season. Excessive rainfall leads to soil flooding (Ye et al., 2018), which affects root respiration (Gupta et al., 2009; Zabalza et al., 2009), nutrient absorption (Gupta et al., 2009; Zabalza et al., 2009), plant morphology (Malik et al., 2002), photosynthesis (Ploschuk et al., 2018; Liu et al., 2020), and energy metabolism and the antioxidant system (Savchenko et al., 2019; Xiao et al., 2020), and ultimately causes a decline in pod yield (Ding et al., 2020; Tian et al., 2020). Besides, with global warming, waterlogging will occur more frequently and undoubtedly have an impact on global food security (Singh, 2015; Yamauchi et al., 2018).

Plant yield is closely related to carbon assimilation and photosynthate distribution. Previous studies have shown that under waterlogging stress, the photosynthetic system in plant leaves was destroyed, and the chlorophyll content was decreased, along with the decrease of the net photosynthetic rate, which might be attributed to the downregulation of genes related to chlorophyll synthesis and the upregulation of genes related to chlorophyll degradation caused by waterlogging (Araki et al., 2012; Ding et al., 2020; Gan et al., 2020; Qi et al., 2020). Studies have shown that, under waterlogging stress, the expressions of genes related to chlorophyll degradation, such as CLH (encoding hydroxymethyl chlorophyll a reductase) and PaO (encoding pheophorbide a oxygenase), were upregulated and that the expressions of genes related to chlorophyll biosynthesis, including HEMB (encoding 5-aminolevulinate dehydrogenase), HEMD (encoding uroporphyrinogen III synthase), and HEMF (encoding coproporphyrinogen III oxidase), were downregulated (Yu et al., 2019). In addition, studies on cotton found that four chlorophyll a/b-binding (LHCBs) genes involved in the light-harvesting complex of photosystem II (PSII) were significantly downregulated by waterlogging stress (Zhang et al., 2017). However, other research revealed that waterlogging could also cause an increase in the net photosynthetic rate, which may be due to the increase of the ethylene content in the plant leaves caused by waterlogging. Additionally, ethylene induced the increase of stomata number or width in plant leaves, which promoted the absorption of carbon dioxide, followed by the increase in the net photosynthetic rate and the dry matter accumulation (Wang et al., 2016; Ceusters and Van de Poel, 2018). In general, the vast majority of studies believe that waterlogging stress leads to the inhibition of photosynthesis of plants, which is not conducive to carbon assimilation in plants.

In addition, peanut plant yield was also related to the effective transformation of photosynthate (Ainsworth and Bush, 2011; Li et al., 2021). Triose phosphate is the main product of photosynthesis and starch is the most common storage form of photosynthate in plants, and photosynthate is mainly transferred from the carbon source to the carbon sink in the form of sucrose (Vu, 2005; Gupta et al., 2017; Ohara and Satake, 2017; Mizuno et al., 2018). The effective transport of photosynthate from source to sink is essential for yield (Julius et al., 2018). However, waterlogging leads to the imbalance of the plant source–sink relationship. Firstly, the starch and sucrose metabolism in the carbon source could be affected by waterlogging stress. A previous study has shown that the accumulation of soluble sugar in the main stem leaf was decreased by waterlogging stress, being consistent with the decreased expression of sucrose metabolism-related genes (Zhang et al., 2017). Moreover, root is disrupted by waterlogging. Under waterlogging stress, soluble carbohydrates in some trees accumulated in the phloem, but the carbohydrate concentration in roots decreased significantly (Kogawara et al., 2006; Merchant et al., 2010). Similarly, it was found that, due to the accumulation of starch in the stem of waterlogged rice, this treatment reduced the distribution rate of assimilation products in the grain (Lee et al., 2019). Waterlogging during grain filling significantly reduced the grain yield of wheat, and the yield losses were related to the decreases in the redistribution of stored photosynthate to the grain and the conversion capacity from carbohydrate to starch in grain (Jiang et al., 2008). In summary, waterlogging resulted in an unbalanced source–sink relationship and a decrease in the effective assimilation of photosynthate to sink, leading to a decrease in yield. However, there is no study on the source–sink relationship of peanut plants under waterlogging stress.

Based on previous studies, the genome sequences of peanut are available (Bertioli et al., 2016, 2019; Chen et al., 2016, 2019; Zhuang et al., 2019), which have facilitated the exploration of the molecular mechanisms and physiological processes in peanuts. At present, the peanut genome sequence has been used to explore the response of peanut to salt stress, the peanut shade avoidance syndrome, and peanut drought tolerance improvement (Cui et al., 2018; Chen et al., 2020; Huang et al., 2020). However, there is no report on the use of transcriptome data to explain the effect of waterlogging on peanut.

To date, most of the research on the effects of waterlogging on the decrease of crop yield is focused on physiology and yield, but there are few studies on the molecular regulation of the photosynthetic characteristics and product distribution that cause yield decline. In this study, the peanut varieties Zhongkaihua 1 (ZKH 1) and Huayu 39 (HY 39) were used. At the flowering stage, the plants of the two peanut varieties were waterlogged for 5 and 10 days, respectively. The aims of this study were to (1) determine the effects of waterlogging on the photosynthetic characteristics and dry matter accumulation of peanut; (2) explore the relationship between starch and sucrose metabolism in leaves, assimilate distribution, and pod yield under waterlogging stress; and (3) identify the differentially expressed genes (*p* < 0.05) under waterlogging stress involved in porphyrin and chlorophyll metabolism, photosynthesis pathways, and starch and sucrose metabolism.

## Materials and Methods

### Plant Material and Growth Conditions

Tetraploid peanut (*Arachis hypogaea* L.) cultivars ZKH 1 and HY 39 were used in the experiment; these two varieties belong to Vulgaris with small peanut kernels. ZKH 1, derived from a crossbreeding between Zhanyou 41 and Yueyou 193, has been widely cultivated in Guangdong Province, China; HY 39, derived from a crossbreeding between Baisha 1016 and Florunner, has been widely grown in Shandong Province, China. The seeds of ZKH 1 and HY 39 are obtained from Zhongkai University of Agriculture and Engineering, Guangdong Province, China, and Peanut Research Institute, Shandong Province, China. In a previous study, we found that HY 39 was more sensitive to waterlogging than ZKH 1 (Zeng et al., 2020). The yield per hectare, the number of total pods, and the number of full pods of HY 39 were more negatively affected by waterlogging than those of ZKH 1. At the same time, the tolerance time to waterlogging of HY 39 was shorter than that of ZKH 1 (Zeng et al., 2020). Seeds of ZKH 1 and HY 39 were grown in plastic pots filled with 35 kg of sandy soil on March 18, 2019, at South China Agricultural University (23°09′30′′N, 113°21′52′′E), in Guangdong Province, China. The size of the pots was 410 mm × 335 mm × 320 mm (top diameter × bottom diameter × height). The sandy soil with pH 5.6 contained 12.2 g kg^–1^ organic matter, 1.1 g kg^–1^ total N, 40.6 mg kg^–1^ available N, 4.5 mg kg^–1^ Olsen-P, and 104.4 mg kg^–1^ available K.

### Waterlogging Treatments

**FIGURE 1:**
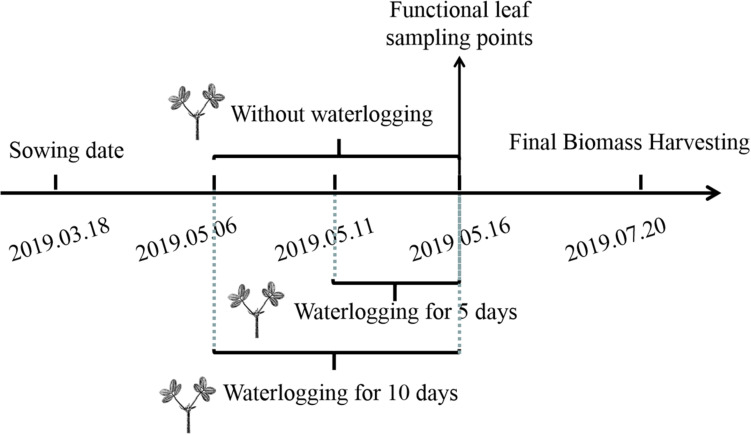
Experimental design.

**FIGURE 2:**
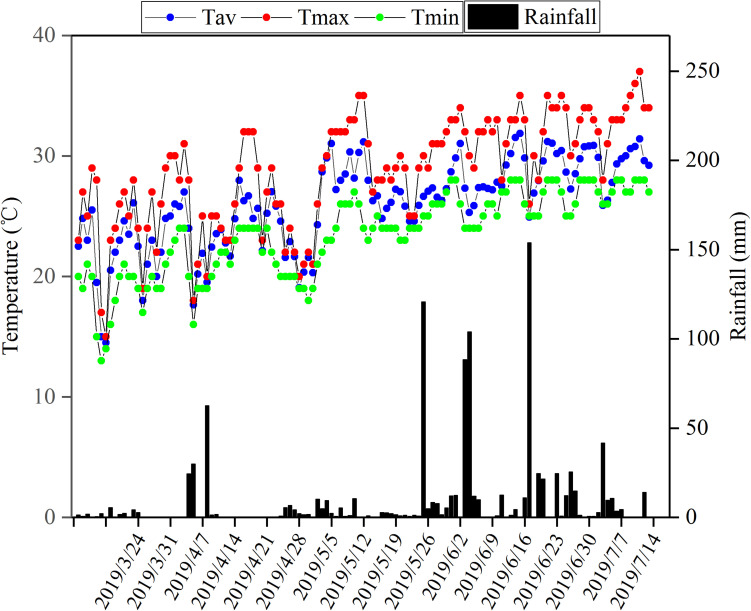
Total rainfall and maximum temperature, minimum temperature, and average everyday temperature during the peanut growing season in Guangzhou, Guangdong, China, in 2019. Tav, average temperature; Tmax, maximum temperature; Tmin, minimum temperature.

During the flowering stage, the peanut plants were treated by waterlogging for 5 and 10 days, respectively, and the control treatment without waterlogging was set (Figure 1). Waterlogging for 10 days: waterlogging started on May 6, 2019 and ended on May 16, 2019; waterlogging for 5 days: waterlogging started on May 10, 2019 and ended on May 16, 2019. The water surface was kept 2 cm above the soil surface. The control plants without waterlogging were watered as normal, with the soil-relative water content (SRWC) = 75%. Soil samples of 0–30 cm from the soil surface were taken by punch every week to determine the fresh weight (FW) and then dried to constant weight in an oven at 80°C to determine the dry weight (DW) of the soil. The saturated soil weight (SW) was estimated by saturating the soil samples. The saturating soil samples and the SRWC were estimated as follows: SRWC = [(FW - DW)/(SW - DW)] × 100. After waterlogging, drain freely and wait for the water content of each barrel to return to the normal level before watering. There were six treatments: (1) Zhongkaihua 1 without waterlogging (Z0); (2) Zhongkaihua 1 with 5 days of waterlogging (Z5); (3) Zhongkaihua 1 with 10 days of waterlogging (Z10); (4) Huayu 39 without waterlogging (H0); (5) Huayu 39 with 5 days of waterlogging (H5); and (6) Huayu 39 with 10 days of waterlogging (H10). Each treatment consisted of 15 pots, and each pot contained two plants. At the end of the waterlogging day (on May 16, 2019), the chlorophyll content and the gas exchange parameters in the third leaf (usually called the functional leaf, positioned from the top downwards) of the main stems of peanut plants from all treatments were measured. Meanwhile, fresh functional leaves were stored in liquid nitrogen at −80°C for RNA sequencing and starch and sucrose metabolism determination. During the growth period, the daily maximum and minimum temperatures were 37 and 13°C, respectively, and the total rainfall was 1,014 mm (Figure 2).

### Determination of Chlorophyll Content and Gas Exchange Parameters

On the day of the end of waterlogging, the chlorophyll content and the gas exchange parameters were measured in the third leaf of the main stems. The chlorophyll content was measured using an SPAD-502 chlorophyll meter (Konica Minolta Sensing Inc., Osaka, Japan). An LI-6400 portable photosynthesis system (LI-COR, Lincoln, NE, United States) with a 6-cm2 leaf area chamber was used to measure the net photosynthetic rate (Pn), intercellular CO2 concentration (Ci), stomatal conductance (gs), and transpiration rate (Tr) in the functional leaves of the main stem. Three representative plants from each treatment were measured at approximately 9:00–11:00 a.m. All measurements were conducted at a photo flux density of 1,400 μmol m-2 s-1 and the ambient CO_2_ concentration of 400 μmol mol-1 at 28°C (Vu, 2005).

### Determination of Starch and Sucrose Metabolism

At the end of the waterlogging treatment, fresh functional leaf samples were frozen in liquid nitrogen and placed in a refrigerator at −80°C to determine the activities of sucrose synthetase (SS) and sucrose phosphate synthetase (SPS). The activities of SPS and SS were determined by the sucrose synthase assay kit (A097) and the sucrose phosphate synthase kit (A098) produced by Nanjing Jiancheng Bioengineering Research Institute. Of the leaf sample, 0.1 g was homogenized by using liquid nitrogen in a pre-chilled mortar and pestle. After homogenization, the sample was extracted with nine times the volume of the extraction buffer and centrifuged at 4,000 rpm for 15 min. According to the manual provided by the manufacturer, the absorption values of SS and SPS were recorded at 290 nm. One unit of SPS and SS is defined as: 1 μmol sucrose production in 1 min by the catalysis in 1 mg tissue at 37°C.

Dry samples (heated under 105°C for 30 min and dried to a constant weight at 80°C) of different tissues of the plants after waterlogging and at the harvest stage were ground to determine the carbohydrates (soluble sugar, sucrose, and starch). Dry samples (0.05 g dry weight) of different tissues were extracted three times with 4 ml of 80% ethanol at 80°C for 30 min. Soluble sugar and sucrose contents were determined in the ethanol supernatant, and starch content was determined from the pellet of the hydroalcoholic extracts (Vega-Mas et al., 2015).

### RNA Extraction, Library Construction, and Sequencing

To explore the molecular mechanisms of waterlogging tolerance in peanut, at the end of waterlogging, the functional leaves were taken for RNA sequencing. Each treatment was replicated three times, with a total of 18 samples. In short, total RNAs were extracted and messenger RNA (mRNA) was enriched to construct a library for sequencing on an Illumina platform HiSeq X Ten in the paired-end 150 bp following prior procedures (Chen et al., 2017). Three replicates collected from each treatment were independently sequenced and 8 Gbp RNA sequencing (RNA-Seq) reads were generated for each sample. The RNA-Seq reads are available at the database Short Read Archive at NCBI^1^. The BioProject and Sequence Read Archive (SRA) accession numbers are PRJNA629848 and SRP259445, respectively.

### Differential Expression Analysis and Functional Enrichment

To obtain high-quality clean reads, the reads were further filtered by Fastp version 0.18.0 and the sequences were aligned to the reference genome (*A. hypogaea* L.). The mapped reads of each sample were assembled using StringTie v1.3.1 in a reference-based approach (Pertea et al., 2015, 2016). For each transcription region, an FPKM (fragment per kilobase of transcript per million mapped reads) value was calculated to quantify its expression abundance and variations using the StringTie software. RNA differential expression analysis between two different groups was performed by DESeq2 software (Robinson et al., 2009; Love et al., 2014). Differentially expressed genes (DEGs) were identified by using DESeq2 with cutoff >twofold changes and *p* < 0.05. To investigate the function and biological pathways involved in the DEGs, we performed Gene Ontology (GO)^2^ and Kyoto Encyclopedia of Genes and Genomes (KEGG)^3^ function enrichment analysis. GO has three ontologies: molecular function, cellular component, and biological process. GO terms, which take the corrected *p* < 0.05 as a threshold, are significantly enriched in DEGs. KEGG pathways with *p* < 0.05 are significantly enriched in DEGs (Moriya et al., 2007).

### Determination of Agronomic and Yield Traits and Dry Matter Accumulation

Three peanut plants were obtained at the harvest stage to determine the agronomic and yield traits and dry matter accumulation. The agronomic traits (including main stem height, lateral branch number, lateral branch length, and stem diameter) were measured and yield traits (number of pods per plant, 100-pod weight, and 100-seed weight) after air drying were recorded. For dry matter accumulation, the leaves, stems, roots, and pods were separated from peanut plants and heated under 105°C for 30 min, dried to a constant weight at 80°C, and weighed, respectively.

### Statistical Analysis

All statistical analyses of the physiological and morphological characteristics were performed with the software package SPSS 22.0 (SPSS, Chicago, IL, United States). All data are the means ± SD of three replicates. Comparisons among multiple groups were performed using the least significant difference (LSD) test. *P* < 0.05 was considered statistically significant.

## Results

### Transcriptome Profiles and Expressional Regulation Responsive to Waterlogging Stress

**FIGURE 3:**
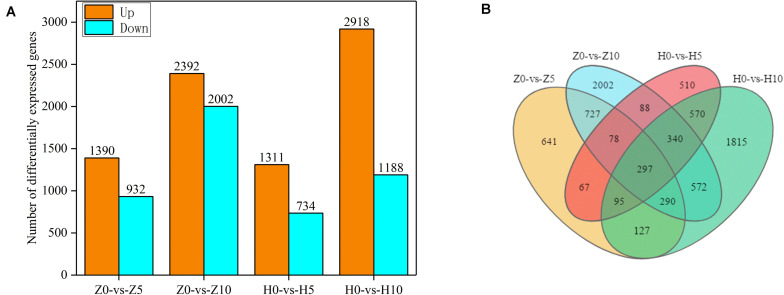
Gene expression of peanut leaves under waterlogging. **(A)** Comparison of the up- and downregulated gene expressions between normal supply and waterlogging (5 and 10 days) in Zhongkaihua 1 (ZKH 1) and Huayu 39 (HY 39). **(B)** Venn diagram of the differentially expressed genes (DEGs) of ZKH 1 and HY 39 under waterlogging. Z0, Zhongkaihua 1 without waterlogging; Z5, Zhongkaihua 1 with 5 days of waterlogging; Z10, Zhongkaihua 1 with 10 days of waterlogging; H0, Huayu 39 without waterlogging; H5, Huayu 39 with 5 days of waterlogging; H10, Huayu 39 with 10 days of waterlogging. The same below. Z0-vs.-Z5, comparison between Zhongkaihua 1 with 5 days of waterlogging (Z5) and Zhongkaihua 1 without waterlogging (Z0); Z0-vs.-Z10, comparison between Zhongkaihua 1 with 10 days of waterlogging (Z10) and Zhongkaihua 1 without waterlogging (Z0); H0-vs.-H5, comparison between Huayu 39 with 5 days of waterlogging (H5) and Huayu 39 without waterlogging (H0); H0-vs.-H10, comparison between Huayu 39 with 10 days of waterlogging (H10) and Huayu 39 without waterlogging (H0).

As shown in Supplementary Table 1, reads were compared with the peanut reference genome. We obtained 395.4845–579.8378 million sequences. The proportion of reads uniquely mapped exceeded 74.68% and that of total mapped reads exceeded 96.77%, which indicated that the sequencing results were of high quality. To identify the DEGs of two peanut varieties, we constructed four libraries under 5- and 10-day waterlogging. The DEGs were identified from pairwise comparisons of the four libraries: Z0-vs.-Z5, Z0-vs.-Z10, H0-vs.-H5, and H0-vs.-H10. A total of 2,322 DEGs were identified in Z0-vs.-Z5, with 1,390 upregulated and 932 downregulated genes. The total number of DEGs in Z0-vs.-Z10 was 4,394, with 2,392 upregulated and 2,002 downregulated genes. Meanwhile, in H0-vs.-H5, a total of 2,045 DEGs were identified, with 1,311 upregulated and 734 downregulated genes. In the case of H0-vs.-H10, 2,918 upregulated and 1,188 downregulated genes were found (Figure 3A). Under the same waterlogging duration, ZKH 1 had more DEGs than HY 39.

In order to identify common and unique DEGs between the two peanut varieties under 5- and 10-day waterlogging stress, we plotted a Venn diagram (Figure 3B). A total of 297 common DEGs were found in these four libraries. Therefore, the transcriptional differences of the DEGs depended on the varieties and the waterlogging duration. At the same time, we found that there were 727 unique DEGs in ZKH 1 and 570 unique DEGs in HY 39 after 5 and 10 days of waterlogging, which might indicate the difference between the two peanut varieties in response to waterlogging.

### Function of Induced DEGs and Affected Pathways by Waterlogging Stress

**FIGURE 4:**
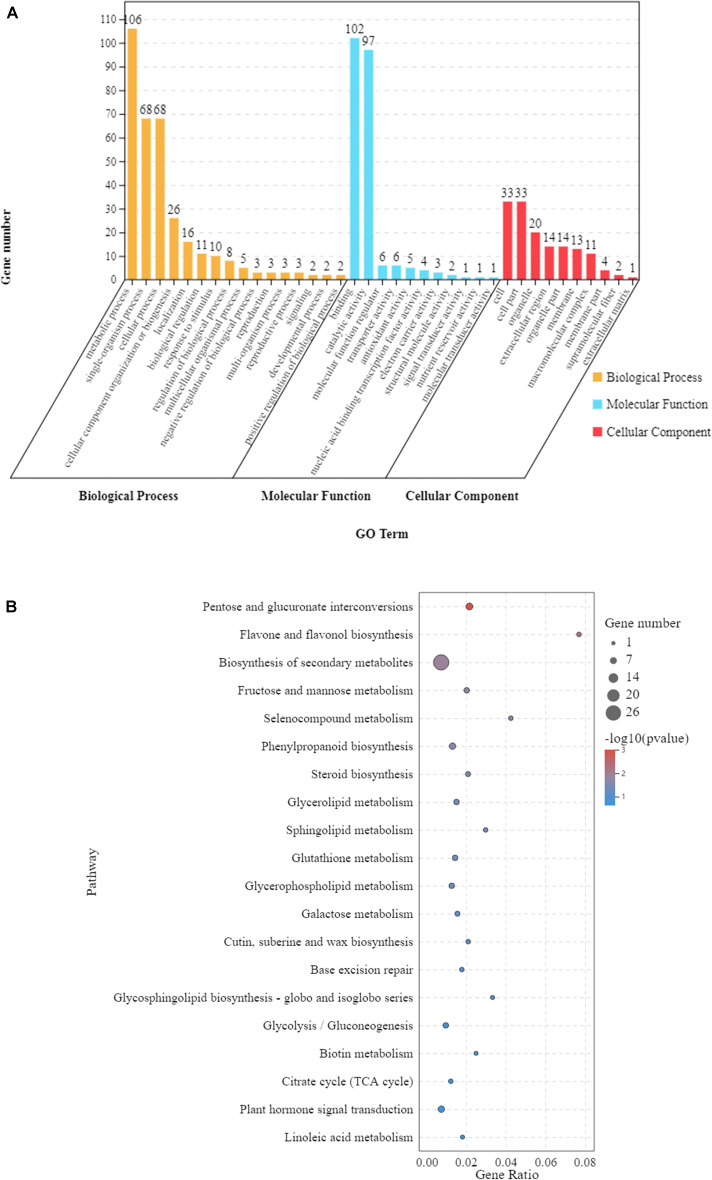
The 297 differentially expressed genes (DEGs) shared by the leaves of Zhongkaihua 1 (ZKH 1) and Huayu 39 (HY 39) under waterlogging for 5 and 10 days. **(A)** Gene Ontology (GO) analysis of the shared genes. The results are summarized into three main categories: biological process, cellular component, and molecular function. The *x*-axis indicates the subcategories and the *y*-axis indicates the number of unigenes. **(B)** Kyoto Encyclopedia of Genes and Genomes (KEGG) analysis of the shared genes. The shared genes were mapped to the KEGG database (see text footnote 3) and the pathway names were extracted. The enrichment was conducted for DEGs involved in each pathway using the genome-scale expressed genes as the background with a hypergeometric test. The first 20 pathways with the smallest *p*-values were used to draw the graph: the ordinate is the pathway and the abscissa is the enrichment factor (the number of differences in the pathway is divided by all the quantities; the size of the bubble represents the quantity, and the redder the color, the smaller the *p*-value).

In order to understand the functions of the 297 DEGs under waterlogging (Figure 3B), we conducted a GO enrichment analysis (hypergeometric test: *q* < 0.05) to classify these DEGs into biological, molecular, and cellular components. These DEGs were enriched in 37 GO terms, among which 16 were related to biological process, 11 were relevant to molecular function, and 10 were associated with cellular component (Figure 4A and Supplementary Table 2). In the biological process category, the main enriched GO terms were metabolic process (GO:0008152), single-organism process (GO:0044699), and cellular process (GO:0009987). In the molecular function category, the main enriched GO terms were binding (GO:0005488) and catalytic activity (GO:0003824). In cellular component, the main enriched GO terms were cell (GO:0005623) and cell part (GO:0044464). At the same time, KEGG pathway analysis showed that these 297 DEGs were mainly involved in 53 pathways, and 10 of them were significantly affected (*p* < 0.05). These pathways were pentose and glucuronate interconversions, flavone and flavonol biosynthesis, biosynthesis of secondary metabolites, fructose and mannose metabolism, selenocompound metabolism, phenylpropanoid biosynthesis, steroid biosynthesis, glycerolipid metabolism, sphingolipid metabolism, and glutathione metabolism (Figure 4B and Supplementary Table 3).

At the same time, in order to better understand the differences between the two peanut varieties in response to waterlogging, we tried to understand the unique DEGs with GO analysis. For ZKH 1 and HY 39, the unique DEGs are mainly concentrated in some GO terms (Supplementary Tables 4, 6), including metabolic process (GO:0008152), single-organism process (GO:0044699), cellular process (GO:0009987), binding (GO:0005488), catalytic activity (GO:0003824), membrane (GO:0016020), cell (GO:0005623), and cell part (GO:0044464). KEGG pathway analysis showed that the unique DEGs of ZKH 1 were mainly focused on flavonoid biosynthesis, circadian rhythm—plant, biosynthesis of secondary metabolites, alpha-linolenic acid metabolism, metabolic pathways, and linoleic acid metabolism (Supplementary Table 5); those in HY 39 were biosynthesis of secondary metabolites and plant hormone signal transduction (Supplementary Table 7).

### Effects of Waterlogging Stress on Porphyrin and Chlorophyll Metabolism in Peanut Leaves

**FIGURE 5:**
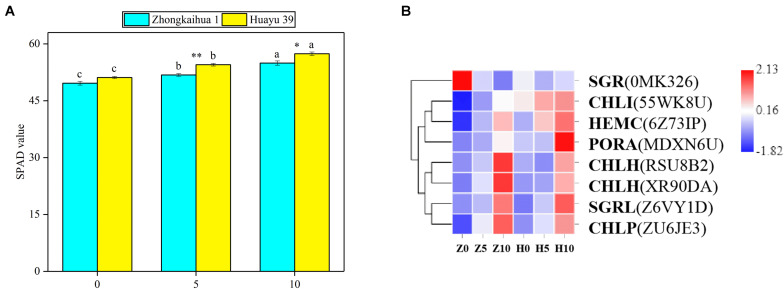
Effects of waterlogging on porphyrin and chlorophyll metabolism. **(A)** Effects of waterlogging stress on the soil and plant analysis development (SPAD) value of peanut at the flowering stage. Data represent the mean ± standard error. The measurement was conducted on the leaves of the main stem after treatment. Letters a and b after the value represent statistically significant differences (*p* < 0.05) within a variety under different waterlogging treatments as determined by the least significant difference test. Significant differences at *p < 0.05 and **p < 0.01 between Zhongkaihua 1 (ZKH 1) and Huayu 39 (HY 39) under the same treatment. 0, 5, and 10 represent without waterlogging, waterlogging for 5 days, and waterlogging for 10 days, respectively. **(B)** Expression patterns of the differentially expressed genes (DEGs) induced by waterlogging stress in the porphyrin and chlorophyll metabolism pathway. Expression change of the DEGs with at least twofold changes and *p* < 0.05 in the porphyrin and chlorophyll metabolism pathway. The expression levels were calculated in fragment per kilobase per million reads (FPKM) from triplicate experiments. Heat map shows the normalized value Z-score after log2(FPKM) transformation. The color in the heat map represents the Z-score after the transformation of log2(mean FPKM). Each row in the heat map represents the levels of a DEG under different conditions. The DEG name and the gene ID in parenthesis were listed on the right side of the heat map. Abbreviations for DEGs encoding proteins: SGR, senescence-inducible chloroplast stay-green protein 2 [Glycine max]; CHLI, magnesium chelatase i2; HEMC, porphobilinogen deaminase; PORA, protochlorophyllide oxidoreductase A; CHLH, magnesium chelatase subunit [Glycine max]; SGRL, senescence-inducible chloroplast stay-green protein 2 [Glycine max]; CHLP, geranylgeranyl diphosphate reductase, chloroplastic [Glycine max].

The effect of waterlogging on the soil and plant analysis development (SPAD) value of peanut leaves is shown in Figure 5A. We found that the SPAD values of the two peanut varieties increased with the extension of the waterlogging time. Under the 5-day waterlogging stress, the SPAD values of ZKH 1 and HY 39 increased by 4.37 and 6.58%, respectively, and those under 10-day waterlogging stress increased by 6.58 and 12.26%, respectively. Under the same waterlogging time, the effect of waterlogging stress on the SPAD value of HY 39 was greater than that of ZKH 1. The changes in the SPAD values may be caused by the changes in gene expression in the porphyrin and chlorophyll pathway.

Eight DEGs were identified in the porphyrin and chlorophyll pathway^4^. We found that genes related to photosynthetic pigment synthesis were upregulated (Figure 5B and Supplementary Table 8), including protochlorophyllide oxidoreductase A (PORA), porphobilinogen deaminase (HEMC), geranylgeranyl diphosphate reductase, chloroplastic [Glycine max] (CHLP), magnesium chelatase i2 (CHLI), and magnesium chelatase subunit [Glycine max] (CHLH). Besides, in senescence-inducible chloroplast stay-green protein 2 [Glycine max], SGRL and SGR are genes concerning chlorophyll degradation; that is, they catalyze chlorophyllide to a pheophytin or a pheophorbide a. SGRL was upregulated while SGR was downregulated.

### Effect of Waterlogging Stress on Photosynthesis of Peanut Leaves

**FIGURE 6:**
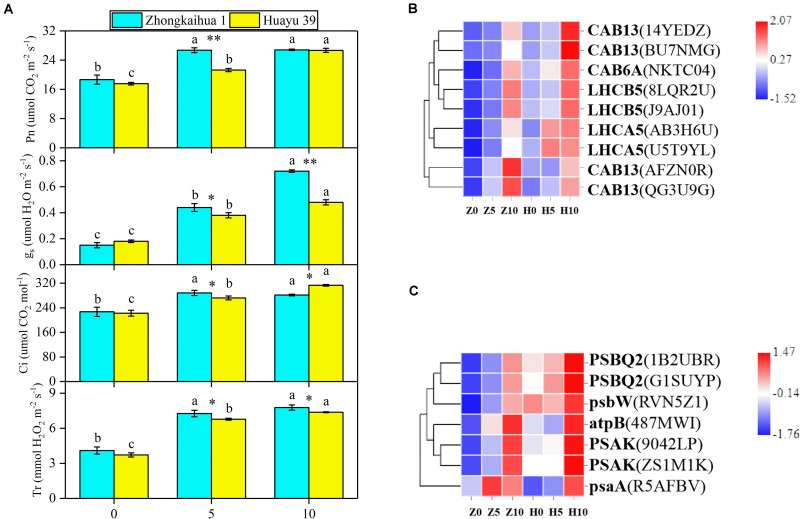
Effects of waterlogging on photosynthesis metabolism. **(A)** Effects of waterlogging stress on the photosynthetic parameters of peanut. Data represent the mean ± standard error. Measurement was conducted on the leaves of the main stem after treatment. Letters a and b after the value represent statistically significant differences (*p* < 0.05) within a variety under different waterlogging treatments as determined by the least significant difference test. Significant differences at *p < 0.05 and **p < 0.01 between Zhongkaihua 1 (ZKH 1) and Huayu 39 (HY 39) under the same treatment. 0, 5, and 10 represent without waterlogging, waterlogging for 5 days, and waterlogging for 10 days, respectively. **(B)** Expression patterns of the differentially expressed genes (DEGs) induced by waterlogging stress in the photosynthesis–antenna proteins pathway. The color in the heat map represents the Z-score after the transformation of log2(mean FPKM). Each row in the heat map represents the levels of a DEG under different conditions. The DEG name and the gene ID in parenthesis were listed on the right side of the heat map. Abbreviations for DEGs encoding proteins: CAB13, light-harvesting chlorophyll B-binding protein 3; CAB6A, light-harvesting chlorophyll B-binding protein 3; LHCB5, light-harvesting chlorophyll B-binding protein 3; LHCA5, light-harvesting chlorophyll B-binding protein 3. Expression change of DEGs with at least twofold changes and *p* < 0.05 in the porphyrin and chlorophyll metabolism pathway. The expression levels were calculated in fragment per kilobase per million reads (FPKM) from triplicate experiments. The heat map shows the normalized value Z-score after log2(FPKM) transformation. **(C)** Expression patterns of the DEGs induced by waterlogging stress in the photosynthesis pathway. Expression change of DEGs with at least twofold changes and *p* < 0.05 in the porphyrin and chlorophyll metabolism pathway. The expression levels were calculated in fragment per kilobase per million reads (FPKM) from triplicate experiments. The heat map shows the normalized value Z-score after log2(FPKM) transformation. The color in the heat map represents the Z-score after the transformation of log2(mean FPKM). Each row in the heat map represents the levels of a DEG under different conditions. The DEG name and the gene ID in parenthesis were listed on the right side of the heat map. Abbreviations for DEG encoding proteins: PSBQ2, oxygen-evolving enhancer protein; psbW, photosystem II reaction center W; atpB, ATP synthase, F1 beta subunit; PSAK, photosystem I reaction center subunit X psaK; psaA, photosystem I P700 chlorophyll A apoprotein.

In order to detect the effect of waterlogging stress on the photosynthesis of peanut leaves, we detected the photosynthesis parameters in the functional leaves of the two varieties and found that, compared with the control group, the Pn, gs, Ci, and Tr of the leaves from plants grown under waterlogging stress for 5 and 10 days increased significantly. However, there were no significant differences in the Pn, Ci, or Tr in ZKH 1 between 10 and 5 days of waterlogging, and gs was 38.89% higher after 10 days of stress than after 5 days. Conversely, there were significant differences in the Pn, gs, Ci, and Tr in HY 39 between 10 and 5 days of waterlogging; these parameters were 25.35, 26.32, 15.07, and 8.85% higher, respectively, after 10 days than after only 5 days of waterlogging (Figure 6A).

We found that nine DEGs, which were enriched in the photosynthesis–antenna proteins pathway^5^, were annotated to code light-harvesting chlorophyll B-binding protein 3 (Figure 6B and Supplementary Table 9). The analysis showed that the expressions of these genes were upregulated under waterlogging, including LHCB5, LHCA5, CAB6A, and CAB13. Additionally, seven DEGs related to the photosynthesis pathway (see text footnote 5) were found (Figure 6C and Supplementary Table 10), all of which were upregulated, including photosystem II reaction center W (psbW), oxygen-evolving enhancer protein (PSBQ2), photosystem I reaction center subunit X psaK (PSAK), photosystem I P700 chlorophyll A apoprotein (psaA), ATP synthase, and F1 beta subunit (atpB). We hypothesized that the upregulation of these genes might be responsible for the increase in Pn.

### Effect of Waterlogging Stress on Starch and Sucrose Metabolism in Peanut Leaves

**FIGURE 7:**
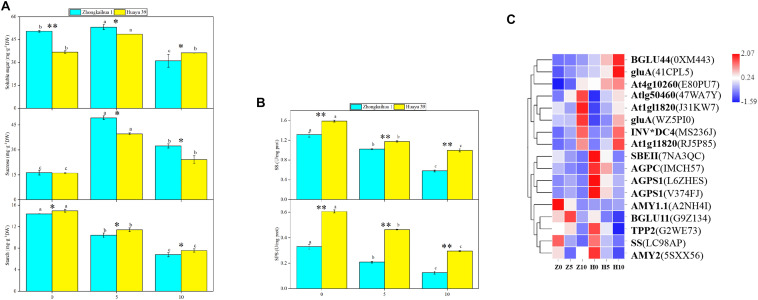
Effects of waterlogging on starch and sucrose metabolism. **(A)** Effects of waterlogging stress on the soluble sugar, sucrose, and starch contents of leaves. Data represent the mean ± standard error. The measurement was conducted on the leaves of the main stem after treatment. Letters a and b after the value represent statistically significant differences (*p* < 0.05) within a variety under different waterlogging treatments as determined by the least significant difference test. Significant differences at *p < 0.05 and **p < 0.01 between Zhongkaihua 1 (ZKH 1) and Huayu 39 (HY 39) under the same treatment. 0, 5, and 10 represent without waterlogging, waterlogging for 5 days, and waterlogging for 10 days, respectively. **(B)** Effects of waterlogging stress on the activities of sucrose synthetase (SS) and sucrose phosphate synthetase (SPS). When determining the enzyme activities, the protein concentrations of homogenized leaf samples from different treatments are shown in Supplementary Figure 3. **(C)** Expression patterns of the differentially expressed genes (DEGs) induced by waterlogging stress in the starch and sucrose metabolism pathway. Expression change of the DEGs with at least twofold changes and *p* < 0.05 in the porphyrin and chlorophyll metabolism pathway. The expression levels were calculated in fragment per kilobase per million reads (FPKM) from triplicate experiments. The heat map shows the normalized value Z-score after log2(FPKM) transformation. The color in the heat map represents the Z-score after the transformation of log2(mean FPKM). Each row in the heat map represents the levels of a DEG under different conditions. The DEG name and the gene ID in parenthesis were listed on the right side of the heat map. Abbreviations for DEG encoding proteins: BGLU44, beta glucosidase 43; gluA, lysosomal beta glucosidase-like isoform X1 [Glycine max]; At4g10260, pfkB-like carbohydrate kinase family protein; At1g50460, hexokinase-like 1; At1g11820, glucan endo-1,3-beta-glucosidase 3-like [Glycine max]; INV*DC4, beta-fructofuranosidase 5; SBEII, 1,4-alpha-glucan-branching enzyme-like [Glycine max]; AGPC, glucose-1-phosphate adenylyltransferase family protein; AGPS1, glucose-1-phosphate adenylyltransferase family protein; AMY1.1, alpha-amylase-like; BGLU11, beta glucosidase 11; TPP2, trehalose-6-phosphate phosphatase; SS, sucrose synthase 4; AMY2, alpha-amylase-like 2.

Compared with the control, under 5 days waterlogging, the contents of soluble sugar and sucrose in the leaves of ZKH 1 increased by 5.47 and 202.65%, respectively, and those of HY 39 increased by 32.09 and 146.92%, respectively. However, under 10 days waterlogging, the content of soluble sugar in the leaves of ZKH 1 and Huayu 39 decreased by 38.48 and 1.22%, respectively, and the content of sucrose increased by 98.23 and 41.82%, respectively (Figure 7A). This may be due to changes in the enzyme activity and related gene expressions in leaves under waterlogging stress. The activities of SS and SPS of the two varieties decreased with the prolongation of the waterlogging time. Among them, 10 days of waterlogging stress had a greater impact on the SS and SPS activities of the two varieties compared to 5 days. The SS and SPS activities of ZKH 1 decreased by 55.88 and 61.81%, respectively, and that of Huayu 39 decreased by 37.18 and 51.35%, respectively, under the 10-day waterlogging treatment (Figure 7B). Ten DEGs were associated with sucrose breakdown, eight {beta-fructofuranosidase 5 (INV^∗^DC4), lysosomal beta glucosidase-like isoform X1 [Glycine max] (gluA), hexokinase-like 1 (At1g50460), pfkB-like carbohydrate kinase family protein (At4g10260), glucan endo-1,3-beta-glucosidase 3-like[Glycine max] (At1g11820)} of which were upregulated and two [(sucrose synthase 4 (SS) and beta glucosidase 11 (BGLU11)] were downregulated (Figure 7C and Supplementary Table 11).

Additionally, the starch content of the leaves of the two varieties decreased with the waterlogging time (Figure 7A), reaching a significant level. Three genes {1,4-alpha-glucan-branching enzyme-like [Glycine max] (SBEII) and glucose-1-phosphate adenylyltransferase family protein (AGPC and AGPS1)} relevant to starch synthesis were downregulated. Alpha-amylase-like 2 (AMY2), a gene correlated with starch breakdown, was downregulated (Figure 7C and Supplementary Table 11).

**FIGURE 8:**
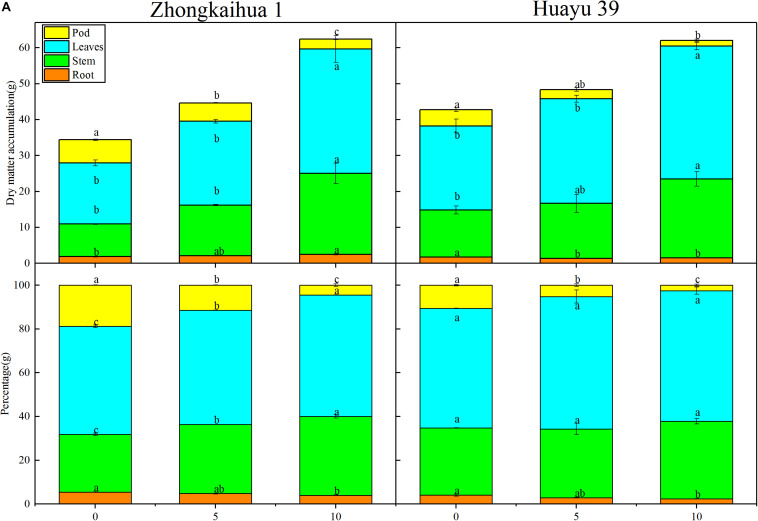
Effects of waterlogging stress on the dry matter accumulation of peanut plant. **(A)** Effects of waterlogging on the dry matter accumulation of the root, stem, leaf, and pod of peanut plant and effects of waterlogging on the percentages of root, stem, leaf, and pod to the total dry matter per plant of peanut. Data represent mean ± standard error. Letters a and b after the value represent statistically significant differences (*p* < 0.05) within a variety under different waterlogging treatments as determined by the least significant difference test.

We speculated that the change of sucrose and starch metabolism in leaves might affect the distribution of photosynthetic products. Therefore, we measured the proportion of plant organs to the total dry matter accumulation (Figure 8) and found that, with the prolongation of the waterlogging time, the leaf dry weight (LDW), stem dry weight (SDW), leaf dry weight to total dry weight (LDW/TDW), and stem dry weight to total dry weight (SDW/TDW) increased, while the pod dry weight (PDW), root dry weight to total dry weight (RDW/TDW), and pod dry weight to total dry weight (PDW/TDW) decreased. The increase in photosynthetic products was contradictory to the decrease of the pod yield. Therefore, we speculated that the translocation and distribution of assimilates are inhibited.

### Effect of Waterlogging Stress on the Morphology and Dry Matter Accumulation of Peanut Plant

**FIGURE 9:**
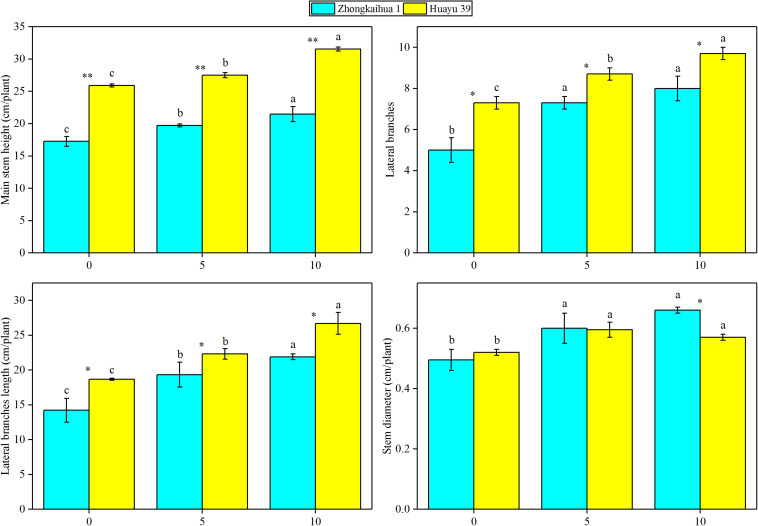
Effects of waterlogging stress on the morphology of peanut plant. Data represent the mean ± standard error. Letters a and b after the value represent statistically significant differences (*p* < 0.05) within a variety under different waterlogging treatments as determined by the least significant difference test. Significant differences at *p < 0.05 and **p < 0.01 between Zhongkaihua 1 (ZKH 1) and Huayu 39 (HY 39) under the same treatment.

The results showed that, at the harvest stage, the main stem height, the number of lateral branches, lateral branch length, stem diameter, TDW, SDW, and LDW of the two varieties increased with increasing duration of the waterlogging treatment. In addition, the RDW of ZKH 1 increased continuously with increasing duration of waterlogging, while the RDW of HY 39 decreased under waterlogging (Figures 8, 9).

### Effect of Waterlogging Stress on the Yield Components of Peanut Plant

**FIGURE 10:**
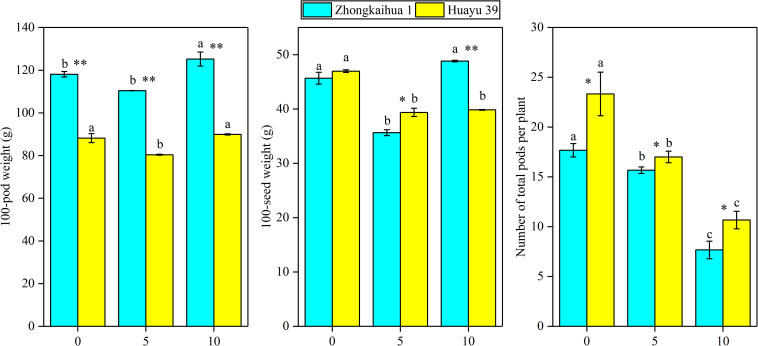
Effects of waterlogging stress on the yield components of peanut plant. Data represent the mean ± standard error. Letters a and b after the value represent statistically significant differences (*p* < 0.05) within a variety under different waterlogging treatments as determined by the least significant difference test. Significant differences at *p < 0.05 and **p < 0.01 between Zhongkaihua 1 (ZKH 1) and Huayu 39 (HY 39) under the same treatment.

It can be seen from Figure 8 that the PDW of the two varieties decreased with the prolongation of the waterlogging time. Under waterlogging for 5 days, the PDW per plant of ZKH 1 and HY 39 decreased by 20.71 and 43.71%, respectively, and under waterlogging for 10 days, the PDW of ZKH 1 and HY 39 decreased by 57.50 and 65.12%, respectively. We can see that, under the same waterlogging time, compared to ZKH 1, the PDW of HY 39 was more susceptible to the impact of waterlogging. Compared with the respective controls, the 100-pod weight, 100-seed weight, and the number of total pods per plant showed a downward trend under waterlogging for 5 days. When exposed to waterlogging for 5 days, the total pods per plant of ZKH 1 and HY 39 decreased by 11.33 and 27.13%, respectively, while those of ZKH 1 and HY 39 under 10 days of waterlogging decreased by 63.82 and 54.26%, respectively. After 10 days of waterlogging treatment, the 100-pod weights of the two varieties were not different from their respective controls. The 100-seed weight of HY 39 was reduced by 15.18%, while that of ZKH 1 was not significantly different from the control (Figure 10).

## Discussion

Waterlogging stress seriously limits the growth and development of plants (Wei et al., 2019; Liu et al., 2020; Ren et al., 2020). With global warming, plants will face a higher risk of waterlogging (Schiermeier, 2011; Kreuzwieser and Rennenberg, 2014; Zhang and Wang, 2019). Previous research showed that waterlogging destroyed the photosynthetic system and reduced the photosynthetic capacity of leaves, mainly reflected in the decrease of the photosynthetic pigment content and Pn, and this is not beneficial to dry matter accumulation (Zeng et al., 2013; Wu et al., 2018). At the same time, the starch and sucrose metabolism of leaves was disordered, and waterlogging inhibited the activities of SS and SPS (Elkelish et al., 2020). Finally, the imbalance of assimilate distribution led to yield penalties (Herzog et al., 2016; Singh et al., 2018). However, studies of waterlogging on plants are mostly focused on the physiological level, but the molecular regulation mechanism behind the yield reduction is unknown (Herzog et al., 2016; Fukao et al., 2019). Here, for the first time, we revealed the systematic effects of waterlogging on peanut morphology, physiology, and molecular regulation, which deepened our understanding of the reasons for yield penalties under waterlogging.

Our study found that waterlogging stress caused the excessive growth of the two peanut varieties and an increase in the dry matter accumulation, which were mainly reflected in the increase of the main stem height, the number of lateral branches, length of lateral branches, and stem diameter. Plant dry matter accumulation is closely related to photosynthesis (Liu et al., 2008; Pérez-Jiménez et al., 2018; Wu et al., 2018; Duan, 2019). However, most previous studies showed that waterlogging led to chlorophyll degradation and photosynthetic system damage, eventually causing a decrease in the SPAD value and the net photosynthetic rate (Ploschuk et al., 2018; Tian et al., 2019). But a study on cotton revealed that 3 days of waterlogging stress increased the net photosynthetic rate of cotton leaves (Wang et al., 2017). However, our study found that the SPAD value and the net photosynthetic rate of the two peanut varieties increased after waterlogging. This may be because short-term waterlogging promoted the stomata opening, which promoted the absorption of carbon dioxide by plants, and the increase of carbon dioxide concentration led to the increase of the net photosynthetic rate of plant leaves (Wang et al., 2016). Similar results were found in wheat. A study found that waterlogging at the grain filling stage improved the leaf photosynthetic capacity of wheat. This may be due to the increase of stomatal conductance and chlorophyll content in leaves under waterlogging, which effectively improved the efficiency of photosynthetic light and carbon use (Lee et al., 2019). In addition, the upregulation of most of the genes involved in the porphyrin and chlorophyll metabolism pathway, photosynthesis–antenna proteins pathway, and the photosynthesis pathway under the condition of waterlogging also confirmed this point, including PORA, HEMC, CHLP, CHLI, CHLH, LHCB5, LHCA5, CAB6A, CAB13, psbW, PSBQ2, PSAK, psaA, and atpB. Therefore, it may be that the upregulation of photosynthesis-related genes caused an improvement in the photosynthetic capacity, which contributed to dry matter accumulation in plants (Fan et al., 2019; Jahan et al., 2020; Zhang et al., 2020).

Previous studies have shown that the increase of photosynthetic capacity promoted plant yield, but our study was contradictory to previous studies. We found that the LDW/TDW and SDW/TDW increased under waterlogging stress, and this finding was consistent with our previous study (Zeng et al., 2020). Therefore, we speculated that the yield loss in this study might be due to the imbalance of the source–sink relationship caused by waterlogging. The photosynthate is transported to storage organs mainly in the form of sucrose via the phloem (Ainsworth and Bush, 2011). In our study, the photosynthate produced by leaves was increased, but did not seem to be effectively transported into the pod. At the same time, an increase of the sucrose and soluble sugar contents and a decrease of the starch content in leaves were observed, which may be due to the downregulation of the SPS and SS enzyme activity. Studies on cotton, wheat, and rapeseed have found that the activities of SS and SPS were decreased under waterlogging (Jiang et al., 2008; Kuai et al., 2014, 2020). Studies showed that stress promoted callose synthesis and precipitation and blocked vascular bundles, and thus reduced the direct transport capacity of vascular bundles to sucrose (Abbasi et al., 2009; Asensi-Fabado et al., 2015), which led to the accumulation of sucrose and soluble sugar contents in plant leaves. Therefore, sucrose accumulation in plant leaves under stress may be caused by the inhibition of sucrose transportation stress. In this research, we detected that two genes associated with sucrose breakdown, including sucrose synthase 4 (SS) and beta glucosidase 11 (BGLU11), were downregulated, which could account for the increase of the sucrose content. Besides, the decrease of the starch content could be attributed to the downregulation of three genes {1,4-alpha-glucan-branching enzyme-like [Glycine max] (SBEII) and glucose-1-phosphate adenylyltransferase family protein (AGPC and AGPS1)} relevant to starch synthesis. The increase of the SDW/TDW may be related to the inhibition of the transport of photosynthate in the stem. A previous study on rice also showed that waterlogging triggers an escape strategy in rice, which is mainly reflected in an increase in the ratio of assimilation product distributed to the stems and a decrease in the ratio of assimilation product distributed to the grain. Increasing the ratio of assimilation products distributed to stems can promote the aboveground growth of crops, which is beneficial to improving the waterlogging tolerance of plants and increasing the survival rate (Lee et al., 2019). In our study, we found that, compared to plants without waterlogging, the soluble sugar content in the stem of the two varieties decreased after waterlogging, and the starch content in the stem of ZKH 1 decreased, while that of HY 39 had no significant difference compared with the control. Interestingly, at the harvest stage, we found that the soluble sugar and starch contents in the stem of the two varieties were higher than those of the control. The sucrose content in leaves increased after waterlogging, but decreased during the harvest stage (Supplementary Figures 1, 2). Therefore, we speculated that the increase of Pn was accompanied by the increase of photosynthate, but the transportation of the photosynthate was blocked and thus accumulated in the stem, which was consistent with the results of previous studies (Camisón et al., 2020; Dingkuhn et al., 2020). There are two possible reasons to account for this. Firstly, the accumulation of soluble sugar and starch in stems probably occurred because the carbon supply by photosynthesis was not synchronized with the carbon demand for functions such as growth and respiration (Sala et al., 2012). This may be because the root was damaged under waterlogging and had less ability to utilize the carbohydrates. Moreover, the decrease in sink demand could also cause the accumulation of sucrose in the stem; in our research, we found that waterlogging decreased the total pod number per plant, which may be a sign of the decrease in sink demand. Secondly, sucrose transportation is regulated by the sucrose transporter, and it is possible that waterlogging led to the downregulation of the protein expression and ultimately led to the inhibition of the transportation of sucrose in the stem (Kühn and Grof, 2010; Geiger, 2011; Saddhe et al., 2020). In general, the accumulation of photosynthate in the stem is probably a strategy for peanut plants to survive under waterlogging.

In terms of the differences between the two cultivars, we analyzed the RNA-Seq results to find out the unique DEGs of the two cultivars under waterlogging. We found that the unique DEGs of the two cultivars were enriched in different pathways, respectively, but these differences seemed to be unrelated to the final yield. By analyzing the PDW per plant at harvest time, we found that, compared to ZKH 1, the PDW of HY 39 decreased more significantly under waterlogging. The changes of the SPAD value and Pn supported the improvement of the photosynthetic characteristics of the two varieties under waterlogging, but it was probably because the photosynthate of HY 39 accumulated more in the stems and leaves than in ZKH1 and transported less into pods (Figure 10), which led to a significant decrease in the yield of HY 39. A previous study revealed that the main reason for the difference in yield between two waterlogged rapeseed varieties was the difference in the carbohydrate translocation from leaves to seeds, which was enhanced in the tolerant variety after waterlogging but inhibited in the sensitive variety; our finding was similar to this (Kuai et al., 2020). Studies on wheat and rice also showed that, under waterlogging stress, the distribution of assimilation products to the stems increased, resulting in an inhibited supply of assimilation products to the grain (Araki et al., 2012; Lee et al., 2019).

In this paper, the reasons for the decrease of peanut yield caused by waterlogging were analyzed from the aspects of porphyrin and chlorophyll metabolism, photosynthesis, and sucrose and starch metabolism. This has some guiding significance for peanut high yield cultivation and waterlogging-tolerant breeding. We found that improvement of the photosynthetic capacity under waterlogging stress increased the dry matter accumulation of plants, but the imbalance of the source–sink relationship was the main cause of yield loss. However, the imbalance of the source–sink relationship is affected by many factors, and it is not enough to explain from the aspect of starch and sucrose metabolism in leaves. We think it necessary to describe the assimilate output from leaves, transportation in stems, and transformation in pods by combining morphology, physiology, and bioinformatics. In addition, the balance of the source–sink relationship is the key point for waterlogging-tolerant breeding and cultivation. To ensure that photosynthetic carbon assimilates of leaves can be transferred to pods, varieties with a harmonious source–sink relationship should be selected under waterlogging. It is important to maintain the balance of the source–sink relationship.

## Conclusion

Under waterlogging stress, the SPAD value and the net photosynthetic rate increased, and the expression of genes related to photosynthesis increased, which indicated that waterlogging enhanced the photosynthetic capacity of plants and was conducive to dry matter accumulation. However, the imbalance of the source–sink relationship under waterlogging was the main reason for the yield loss. Waterlogging led to the disorder of starch and sucrose metabolism in leaves and the photosynthate accumulation in stems and leaves. The reason why the yield of HY 39 decreased more than that of ZKH 1 may be that more proportion of the photosynthate accumulated in the stems and leaves of HY 39 and could not be effectively transported to the pod.

## Data Availability Statement

Publicly available datasets were analyzed in this study. This data can be found here: The RNA-Seq reads are available at the database Short Read Archive at NCBI (https://www.ncbi.nlm.nih.gov). The BioProject and Sequence Read Archive (SRA) accession are PRJNA629848 and SRP259445, respectively.

## Author Contributions

RZ, TC, JZ, and LZ conceptualized the study, wrote the original draft preparation, supervised, and administered the project. TC, XW, JC, XL, XX, QX, YD, LH, LW, and LZ contributed to the methodology. XW, JC, XL, XX, QX, YD, LH, JZ, and LZ helped with software. JZ and LZ did the validation, did the writing, review, editing, and helped with funding acquisition. TC, JZ, and LZ did the formal analysis. RZ, JZ, and LZ contributed to the investigation and contributed to the visualization. RZ, TC, and XW helped with resources and curated the data. All authors contributed to the article and approved the submitted version.

## Conflict of Interest

The authors declare that the research was conducted in the absence of any commercial or financial relationships that could be construed as a potential conflict of interest.

## Footnotes

1
https://www.ncbi.nlm.nih.gov

2
http://geneontology.org/

3
http://www.genome.jp/kegg/

4
https://www.genome.jp/kegg/pathway/map/map00860.html

5
https://www.genome.jp/kegg/pathway/map/map00196.html
