# Corrigendum: Physiological and Expressional Regulation on Photosynthesis, Starch and Sucrose Metabolism Response to Waterlogging Stress in Peanut

**DOI:** 10.3389/fpls.2021.783044

**Published:** 2021-10-19

**Authors:** Ruier Zeng, Tingting Chen, Xinyue Wang, Jing Cao, Xi Li, Xueyu Xu, Lei Chen, Qing Xia, Yonglong Dong, Luping Huang, Leidi Wang, Jialei Zhang, Lei Zhang

**Affiliations:** ^1^College of Agriculture, South China Agricultural University, Guangzhou, China; ^2^Bio-Tech Research Center, Shandong Academy of Agricultural Science, Jinan, China

**Keywords:** *Arachis hypogaea* L., waterlogging stress, photosynthesis, starch and sucrose metabolism, yield

In the published article, there was an error regarding the affiliation of the author Lei Zhang. This author's affiliation was incorrectly presented as “Bio-Tech Research Center, Shandong Academy of Agricultural Science, Jinan, China”; the correct affiliation should be “College of Agriculture, South China Agricultural University, Guangzhou, China.”

The authors apologize for this error and state that this does not change the scientific conclusions of the article in any way. The original article has been updated.

## Publisher's Note

All claims expressed in this article are solely those of the authors and do not necessarily represent those of their affiliated organizations, or those of the publisher, the editors and the reviewers. Any product that may be evaluated in this article, or claim that may be made by its manufacturer, is not guaranteed or endorsed by the publisher.

