# Supplementary material for: Physiological and Expressional Regulation on Photosynthesis, Starch and Sucrose Metabolism Response to Waterlogging Stress in Peanut
Source: Front Plant Sci. 2021 Jul 2;12:601771. doi: 10.3389/fpls.2021.601771 (PMC8283264; doi:10.3389/fpls.2021.601771)
Supplement: Supplementary file 5 [file Data_Sheet_1.docx]

Supplementary Material

# Supplementary Tables

**Supplementary Table S1.** Table S1 Statistics of raw reads and comparison with peanut genome

**Supplementary Table S2.** GO enrichment analysis of 297 shared DEGs (logFC>2 and FDR < 0.05) in Zhongkaihua 1 and Huayu 39 under waterlogging for 5 and 10 days

**Supplementary Table S3.** KEGG analysis of 297 snared DEGs (logFC>2 and FDR < 0.05) in Zhongkaihua 1 and Huayu 39 under waterlogging for 5 and 10 days

**Supplementary Table S4.** GO enrichment analysis of 727 unique DEGs (logFC>2 and FDR < 0.05) in Zhongkaihua 1 (ZKH1) under waterlogging for 5 and 10 days

**Supplementary Table S5.** KEGG enrichment analysis of 727 unique DEGs (logFC>2 and FDR < 0.05) in Zhongkaihua 1 (ZKH1) under waterlogging for 5 and 10 days

**Supplementary Table S6.** GO enrichment analysis of 570 unique DEGs (logFC>2 and FDR < 0.05) in Huayu 39 (HY 39) under waterlogging for 5 and 10 days

**Supplementary Table S7.** KEGG enrichment analysis of 570 unique DEGs (logFC>2 and FDR < 0.05) in Huayu 39 (HY 39) under waterlogging for 5 and 10 days

**Supplementary Table S8.** The shared DEGs related with porphyrin and chlorophyll metabolism

**Supplementary Table S9.** The shared DEGs related with photosynthesis-antenna proteins pathway

**Supplementary Table S10.** The shared DEGs related with photosynthesis

**Supplementary Table S11.** The shared DEGs related with starch and sucrose metabolism

# Supplementary Figures

**Supplementary Figure S1.** Effects of waterlogging stress on soluble sugar, sucrose, starch content of root and stem after waterlogging. Data represent mean ± standard error. The measurement was conducted on leaves on the main stem after a treatment. Letters a, b after the value represent the statistically significant difference (*p* < 0.05) within a variety under different waterlogging treatments as determined by the Least Significant Difference test. * represent a significant difference at *p* < 0.05 between Zhongkaihua 1 (ZKH 1) and Huayu 39 (HY 39) under the same treatment, and ** represent a significant difference at *p* < 0.01 between Zhongkaihua 1 (ZKH 1) and Huayu 39 (HY 39) under the same treatment. 0, 5 and 10 represent without waterlogging, waterlogging for 5 days, waterlogging for 10 days, respectively.

**Supplementary Figure S2.** Effects of waterlogging stress on soluble sugar, sucrose, starch content of root, stem leaves and pods at the harvest stage. Data represent mean ± standard error. The measurement was conducted on leaves on the main stem after a treatment. Letters a, b after the value represent the statistically significant difference (*p* < 0.05) within a variety under different waterlogging treatments as determined by the Least Significant Difference test. * represent a significant difference at *p* < 0.05 between Zhongkaihua 1 (ZKH 1) and Huayu 39 (HY 39) under the same treatment, and ** represent a significant difference at *p* < 0.01 between Zhongkaihua 1 (ZKH 1) and Huayu 39 (HY 39) under the same treatment. 0, 5 and 10 represent without waterlogging, waterlogging for 5 days, waterlogging for 10 days, respectively.

**Supplementary Figure S3.**  The protein concentration of homogenized leaf samples from different treatments. Data represent mean ± standard error. The measurement was conducted on leaves on the main stem after a treatment. Letters a, b after the value represent the statistically significant difference (*p* < 0.05) within a variety under different waterlogging treatments as determined by the Least Significant Difference test. * represent a significant difference at *p* < 0.05 between Zhongkaihua 1 (ZKH 1) and Huayu 39 (HY 39) under the same treatment, and ** represent a significant difference at *p* < 0.01 between Zhongkaihua 1 (ZKH 1) and Huayu 39 (HY 39) under the same treatment. 0, 5 and 10 represent without waterlogging, waterlogging for 5 days, waterlogging for 10 days, respectively.

**
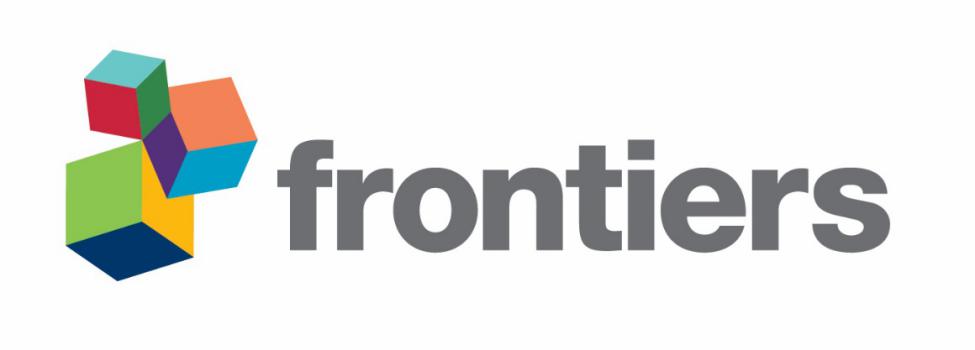
**
